# Anaerobic decomposition of humic substances by *Clostridium* from the deep subsurface

**DOI:** 10.1038/srep18990

**Published:** 2016-01-08

**Authors:** Akio Ueno, Satoru Shimizu, Shuji Tamamura, Hidetoshi Okuyama, Takeshi Naganuma, Katsuhiko Kaneko

**Affiliations:** 1 Horonobe Research Institute for the Subsurface Environment, Northern Advancement Centre for Science and Technology, 5-3, Sakae-machi, Horonobe-cho, Teshio-gun, Hokkaido 098-3221, Japan; 2 Graduate School of Environmental Earth Science, Hokkaido University, Sapporo, Hokkaido 060-0810, Japan; 3 Graduate School of Biosphere Science, Hiroshima University, Higashi-Hiroshima 739-8528, Japan

## Abstract

Decomposition of humic substances (HSs) is a slow and cryptic but non-negligible component of carbon cycling in sediments. Aerobic decomposition of HSs by microorganisms in the surface environment has been well documented; however, the mechanism of anaerobic microbial decomposition of HSs is not completely understood. Moreover, no microorganisms capable of anaerobic decomposition of HSs have been isolated. Here, we report the anaerobic decomposition of humic acids (HAs) by the anaerobic bacterium *Clostridium* sp. HSAI-1 isolated from the deep terrestrial subsurface. The use of ^14^C-labelled polycatechol as an HA analogue demonstrated that the bacterium decomposed this substance up to 7.4% over 14 days. The decomposition of commercial and natural HAs by the bacterium yielded lower molecular mass fractions, as determined using high-performance size-exclusion chromatography. Fourier transform infrared spectroscopy revealed the removal of carboxyl groups and polysaccharide-related substances, as well as the generation of aliphatic components, amide and aromatic groups. Therefore, our results suggest that *Clostridium* sp. HSAI-1 anaerobically decomposes and transforms HSs. This study improves our understanding of the anaerobic decomposition of HSs in the hidden carbon cycling in the Earth’s subsurface.

The carbon mass in the Earth’s crust, including sediments and sedimentary rock, is estimated to be 7.78–9.00 × 10^22^ g C, which is second only to the carbon pool in the mantle^1^. Although the majority of crustal carbon is inorganic and lies in limestone, the organic carbon mass is estimated to be 1.25 × 10^22^ g C^1^, corresponding to approximately one sixth of the crustal carbon. Measurements of contemporary sediments indicate that refractory fractions, or humic substances (HSs), account for 40–90% of the total organic carbon in sediments^2^.

To reveal the global carbon cycle through the microbial decomposition of HSs in the sedimentary rock systems, we focused on the anaerobic decomposition of HS. This study reports the anaerobic decomposition of HSs by a single bacterium isolated from the deep subsurface, rather than by a microbial consortium. The bacterium decomposed both commercial and natural humic acids (HAs) extracted from the deep subsurface diatomite layer in the northernmost region of Hokkaido, Japan^3^. Given that the bacterium and HAs were collected from subsurface environments, anaerobic HS decomposition may occur widely *in situ*. Most of the decomposition products were found in lower molecular mass fractions as expected.

## Results

### Isolation and growth properties of an HS-decomposing bacterium

We isolated a strictly anaerobic HS-decomposing bacterium following enrichment cultivation in media containing commercial HSs (Aldrich HAs). The 16S rRNA gene sequence of the isolate was most closely related to that of *Clostridium puniceum* DSM 2619^T^ (98.9% similarity) (Supplementary Fig. 1); therefore, the isolate was designated *Clostridium* sp. strain HSAI-1.

The growth of strain HSAI-1 was monitored based on the increase in cell numbers in media containing Aldrich HAs or naturally occurring HAs from the Koetoi diatomite layer with or without 0.5% glucose (Supplementary Table 1). The initial cell density in each experiment was set at 1.2–1.5 × 10^7^ cells mL^−1^. A 10-fold increase occurred in 8 days in cultures supplemented with glucose, whereas only a small increase was observed when HAs were the sole carbon source. In subsequent experiments, cultures were supplemented with 0.5% glucose.

### Decomposition of a ^14^C-labelled HA analogue

**Figure 1:**
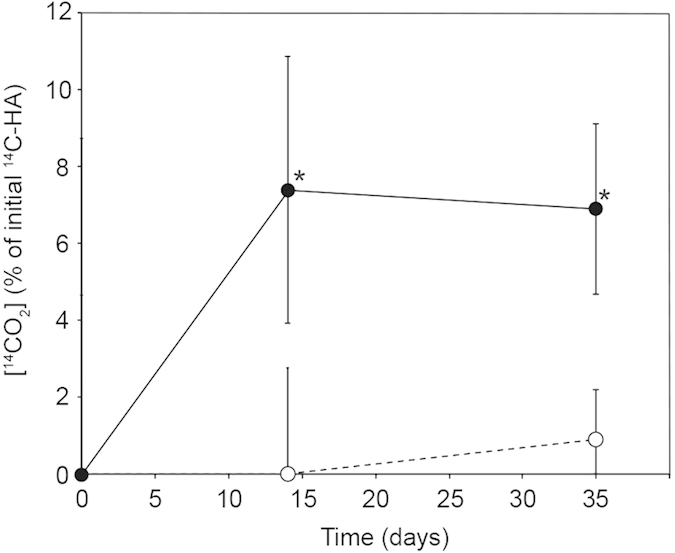
^14^CO_2_ evolution in an anaerobic culture of *Clostridium* sp. strain HSAI-1. The ^14^CO_2_ in the headspace gas was measured via liquid scintillation counting of inoculated cultures (filled circles). Uninoculated control cultures (open circles) yielded little or no ^14^CO_2_. The data points are the mean values of triplicate samples ± standard deviations. *P < 0.05 was considered significant in Student’s *t*-test.

The release of ^14^CO_2_ from ^14^C-labelled polycatechol, an HA analogue, was monitored as an index of bacterial HA decomposition (Fig. 1). Maximal ^14^CO_2_ evolution corresponding to 7.4 ± 3.5% of the exogenous ^14^C-HA was observed in cultures containing strain HSAI-1 in the first 14 days, compared with little or no ^14^CO_2_ evolution in the uninoculated control experiments (*P* < 0.05). The rate of ^14^CO_2_ evolution slowed thereafter, likely due to the increased recalcitrance of polycatechol to biodegradation. The total balance of ^14^C was calculated at the end of this experiment (Table 1). In the uninoculated control experiments, a small amount of ^14^CO_2_ (0.7%) was detected, which was attributed to spontaneous release, and 58.5% soluble and 40.8% insoluble fractions were retrieved. Conversely, experiments inoculated with HSAI-1 yielded 6.6% ^14^CO_2_ (approximately 10-fold the amount obtained through “spontaneous” release); in this case, 43.0% soluble and 50.4% insoluble fractions were retrieved. The proportion of insoluble fractions in the experiments with HSAI-1 was higher than those in the uninoculated controls.

### Instrumental analyses of HAs

**Figure 2:**
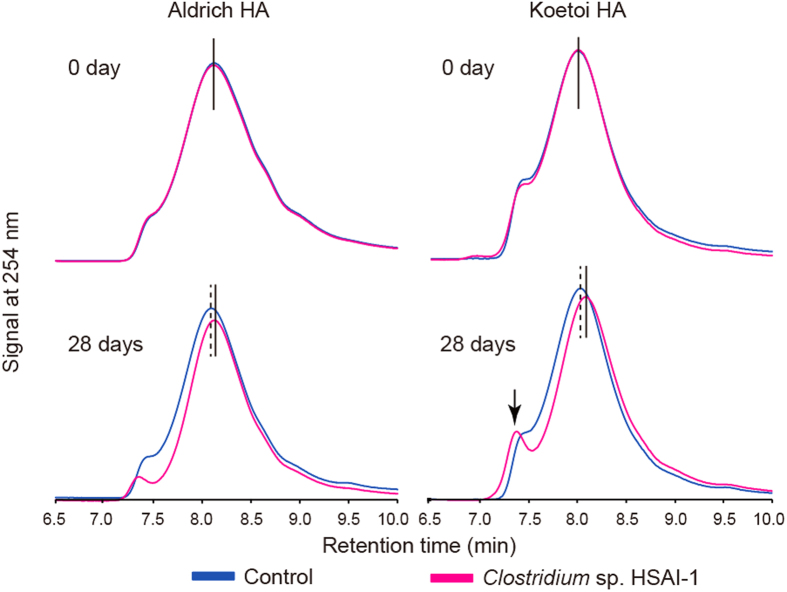
HSs are decomposed anaerobically by *Clostridium* sp. strain HSAI-1. Left column, chromatograms of Aldrich HAs at 0 and 28 days. Right column, chromatograms of Koetoi HAs at 0 and 28 days. Each experimental group consisted of 5 culture setups (*n* = 5). One representative chromatogram from each experimental group is shown. For clarity, chromatograms from uninoculated control cultures are depicted in blue, and those from inoculated cultures (using strain HSAI-1) are shown in magenta. The tops of the main peaks with retention times ranging from 7.8 to 8.3 min are indicated with vertical lines: a dotted line for the uninoculated controls and a solid line for the inoculated cultures. The peak indicated by the downward-pointing arrowhead corresponds to the high-molecular mass HAs in cultures inoculated with strain HSAI-1.

Using high-performance size-exclusion chromatography (HPSEC), we demonstrated the decomposition of HAs during a 28-day incubation with the *Clostridium* sp. strain HSAI-1. HA decomposition was confirmed in terms of its amounts and molecular masses. While decreased peaks indicate decreased HA levels, delays in retention times reflect reductions in molecular mass. Major chromatographic peaks at retention times of 7.3–8.3 min, which corresponded to molecular masses of approximately 2-3 kDa, indicated reductions in molecular mass (Fig. 2 and Supplementary Fig. 4). The mean values ( ± SD) of the molecular masses at the peaks were calculated and are shown in Table 2. At day 0, significant differences were not observed between the uninoculated control and the HSAI-1 inoculated culture (2790.1 ± 15.5 vs. 2801.4 ± 12.7 Da for Aldrich HAs, *P* = 0.242; 3317.9 ± 0.0 vs. 3286.2 ± 31.7 Da for Koetoi HAs, *P* = 0.056), whereas a significant difference was observed at 28 days (2887.3 ± 12.9 vs. 2673.1 ± 76.8 Da for Aldrich HAs, *P* < 0.001; 3229.6 ± 14.0 vs. 2824.4 ± 51.8 Da for Koetoi HAs, *P* < 0.001) (Table 2). The reductions were 214.2 Da for the commercial Aldrich HAs and 405.2 Da for the natural Koetoi HAs. Similar shifts in peak height and retention time were reproduced with a well-known aerobic HA-degrading actinomycete, *Streptomyces viridosporus* (ATCC 39115; Supplementary Fig. 3)^4^. To our surprise, a conspicuous peak at 7.3 min in the Koetoi HAs increased in both its amount and molecular mass (Fig. 2, lower right); the molecular mass of the peak exceeded 50 kDa, as estimated based on the size exclusion limit of the column.

**Figure 3:**
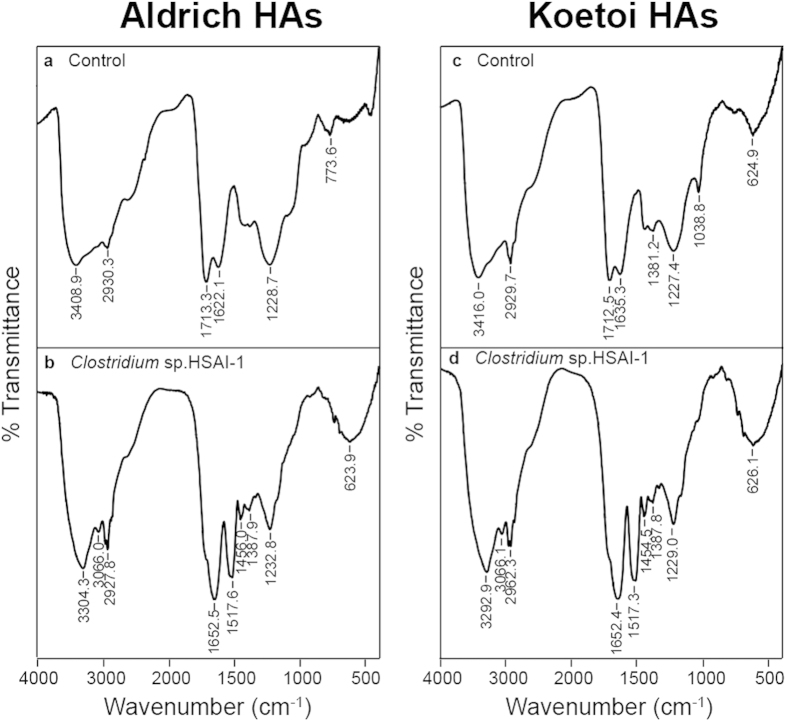
FT-IR spectra of HAs. Left column, Aldrich HAs. Right column, Koetoi HAs. Representative FT-IR spectra are shown for Aldrich HAs or Koetoi HAs (*n* = 3). (**a,c)** Uninoculated control cultures. (**b,d)** Cultures inoculated with *Clostridium* sp. strain HSAI-1.

Through Fourier transform infrared (FT-IR) analysis, we demonstrated that the bacterial HA decomposition induced structural changes in HAs (Fig. 3 and Supplementary Table 2). Several notable changes can be summarised as follows: 1) the disappearance of two absorption bands in Aldrich HAs and three bands in Koetoi HAs, specifically, the bands near 3410–3420 and 1710 cm^−1^ in both HAs and one near 1035 cm^−1^ in Koetoi HA; and 2) the appearance of four absorption bands near 3290–3300, 3070, 1540 and 1450 cm^−1^ in both HAs. The absorption bands near 2930, 1650 and 1230–1240 cm^−1^ were unchanged in both HAs. The absorption band near 3410–3420 cm^−1^ was assigned to H-bonded OH groups, and the absorption band near 1710 cm^−1^ was assigned to C=O stretching vibrations in carboxyls, aldehydes and ketones^5^. The absorption band near 1035 cm^−1^ in the Koetoi HAs was assigned to alcoholic and polysaccharide C–O stretching vibrations and OH deformations^6^. These results suggest bacterial removal of carboxyl groups and polysaccharide-related moieties and the addition of aliphatic structural units, amide groups, and aromatic groups.

## Discussion

Our study suggests the possibility that HSs are microbially decomposed in the deep subsurface environment, where aerobic conditions are unlikely. Aerobic decomposition of HSs by microorganisms in the surface environment has been well documented^4^,^7^,^8^,^9^,^10^,^11^,^12^. However, the biogeochemical importance of the anaerobic decomposition of HSs remains poorly understood, although HSs are known to play an important role in the global carbon cycle^13^,^14^,^15^,^16^. Our results clearly showed that a single bacterial isolate under strictly anaerobic conditions can decompose HSs. Changes in HA structures during decomposition were previously observed in HA-containing fertilisers supplemented with steel slag as a source of soluble Fe(II)^17^. This previous study assumed the involvement of undefined sulfate-reducing bacteria, whereas our study focused on pure cultures of a *Clostridium* species.

Biopolymers, such as cellulose, lignocellulose and lignin, are major components of biomass in nature and account for approximately half of the matter produced by photosynthesis^18^. Clostridia can hydrolyse biopolymers, ferment monomers and amino acids, and produce alcohols, organic acids and hydrogen^19^. In particular, the best-studied anaerobic bacterium, *C. thermocellum,* hydrolyses lignocellulose to ferment the resulting sugars directly into ethanol^20^. Since strain HSAI-1 is classified into the genus *Clostridium* (Supplementary Fig. 1), this bacterial strain may be able to degrade biopolymers under anaerobic conditions in the context of the previously mentioned studies.

The deep terrestrial subsurface environment is generally thought to be oligotrophic. In this study, glucose supplementation facilitated the decomposition of HSs, and we demonstrated that strain HSAI-1 has potential to decompose HSs under anaerobic conditions. This setting would be unlikely in the deep subsurface environment; however, a recent theory has proposed that soil organic carbon in deep soil layers that has escaped microbial degradation and remained as ancient organic carbon for more than several thousand years could be subject again to degradation by microorganisms if an appropriate fresh organic carbon such as cellulose were to be supplied^21^. If anaerobic bacteria are stimulated by assimilable organic carbon existing in the subsurface environment, then the decomposition of HSs might occur in the deep subsurface environment.

Historically, HSs have been operationally defined as humic and fulvic acids and humin based on their solubility under acidic or alkaline conditions^13^. However, recent information has shown that HSs are a mixture of diverse and relatively low molecular mass components. Direct *in situ* observations have demonstrated the formation of such associations between relatively small molecules by hydrophobic interactions and hydrogen bonds^22^,^23^. Nevertheless, the fundamental molecular structure of HSs is assumed to be similar to lignin because lignin is a major parent material in the formation of HSs^24^. Colberg and Young identified degradation products of lignin in a methane-forming consortium^25^, and they also identified 10 monomeric compounds including phenylacetate, benzoate, catechol, 3-phenylpropionate, cinnamate, *p*-hydroxybenzoate, *p*-hydroxycinnamate, syringate, vanillate, ferulate, caffeinate and vanillin^25^. Therefore, the anaerobic degradation of HA by strain HSAI-1 might produce monoaromatic compounds as degradation products of the HAs. Determining the compounds that are produced after the decomposition of HAs will be the focus of a future study.

It is widely accepted that HSs act as terminal electron acceptors under anaerobic conditions^26^,^27^,^28^,^29^,^30^,^31^. Nevertheless, coupling of HS reduction and HS degradation (such as decrease in molecular mass) has not been experimentally manifested. In our study, glucose was added to the culture media as a second carbon source that is easy to assimilate to strain HSAI-1. Under this condition, HAs were assumed to be used as terminal electron acceptors in the presence of glucose. Although the HA reduction should be taken into account, we could not determine whether HA was utilized as an electron acceptor. Another possibility would be the degradation of HA caused by co-metabolism. The white rot fungus *Phanerochaete chrysosporium* is capable of degrading HAs with the addition of glucose; nevertheless, *P. chrysosporium* did not utilize HS as the sole carbon source^32^.

Difference of FT-IR spectral patters between the uninoculated control and the HSAI-1 inoculated culture clearly demonstrated the decomposition of HA. Especially, the disappearance of the bands near 1710 cm^−1^ in both HAs and 1035 cm^−1^ in the Koetoi HAs was consistent with the result of a previous study in which the C=O stretch from COOH at 1711 cm^−1^ and the polysaccharide-related IR band at 1033 cm^−1^ disappeared from the spectrum almost completely because of microbial degradation^33^,^34^. Our results also showed that ^14^CO_2_ was released into the headspace during incubation when ^14^C-labelled polycatechol was used as substrate (Fig. 1 and Table 1). Taken together, these results indicated that carboxyl groups in both the Aldrich and Koetoi HAs and alcohol or polysaccharide-related substances in the Koetoi HAs are decomposed by strain HSAI-1, and CO_2_ is released, thus supporting the possibility of HA decomposition.

Previous studies revealed that lignolytic fungi degrade HAs concomitantly with the increasing amount of insoluble HAs in fungal cultures, which are not extractable with NaOH^7^,^8^. The authors suggested that the fungi not only degrade HAs but also produce refractory humin^8^. Some of the insoluble fraction in experiments inoculated with HSAI-1 may have been incorporated into biomass or closely bound to the cell surface, thus forming the refractory humic fraction. The production of acid-precipitable polymeric lignin with a molecular mass of >20 kDa during lignin degradation by *S. viridosporus* has been previously reported^35^. Similar substances may have also been produced in our *Clostridium* cultures.

In conclusion, we confirmed the decomposition of HSs by pure cultures of a strictly anaerobic bacterium isolated from the deep terrestrial subsurface. This finding conflicts with the previously reported decomposition of lignin, an HS component, by mixed cultures of soil microorganisms^36^,^37^. We supplemented the cultures with glucose to facilitate HS decomposition based on the hypothesis that supplies of fresh carbon may induce the microbial decomposition of organics that are stable over the long term in deep soil^21^. With this new knowledge, the biodegradation of persistent organic matter in the deep subsurface can be better defined, predicted and controlled.

## Methods

### Chemicals and bacterial strains

Aldrich humic acids (Aldrich HAs), in the form of humic acid sodium salt, were purchased from Sigma-Aldrich (St. Louis, MO, USA). HA-degrading *S. viridosporus* ATCC 39115 was purchased from ATCC (American-Type Culture Collection, Manassas, VA, USA) and was used as the reference strain^16^. *Clostridium* sp. strain HSAI-1 was deposited at DSMZ (Deutsche Sammlung von Mikroorganismen und Zellkulturen GmbH, Braunschweig, Germany) and NBRC (NITE Biological Resource Center, National Institute of Technology and Evaluation, Kisarazu, Japan) under the accession numbers of DSM 100957 and NBRC 111506, respectively.

### Sample collection

Groundwater samples were collected independently from three sites: the Horonobe deep borehole (HDB) in the Horonobe Underground Research Laboratory, the Yubari coal-bed methane recovery site, and a gas-petroleum reservoir in Higashi-Niigata, Japan (where mesothermic production water was collected). The geological features and the characteristics of archaeal and bacterial community structures in those areas were described previously^38^,^39^,^40^. The samples were stored in sterilised 1-L polypropylene bottles and transferred immediately to our laboratory. The mixture of groundwater collected from the three sites was used for the enrichment and cultivation of methanogens. After several transfers, an aliquot of the enriched culture was used as the inoculum for the enrichment and cultivation of HA-degrading bacteria. Siliceous mudstone samples were collected from the Koetoi Formation, which is composed of diatomite from the Miocene to Pliocene epochs, and used for preparing HAs as described below.

### Preparation and extraction of HAs

The preparation of Aldrich and Koetoi HAs and HA extraction from culture media were performed according to the methods described by the International Humic Substances Society^41^. Throughout the study, HA-containing solutions were filter-sterilised using membrane filtration units (pore size 0.22 μm) before addition to culture media.

### Media preparation and culturing techniques

The LPB culture medium was prepared with a slight modification in Hungate tubes (Chemglass Life Sciences, Vineland, NJ, USA) and was used as a basal liquid medium for the enrichment, isolation and maintenance of HSAI-1^42^. The medium contained the following (per litre of distilled water): KCl, 0.1 g; MgSO_4_•7H_2_O, 4.0 g; NH_4_Cl, 0.5 g; CaCl_2_•2H_2_O, 0.14 g; K_2_HPO_4_, 0.14 g; NaCl, 6.0 g; Fe(NH_4_)_2_(SO_4_)_2_•6H_2_O, 2 mg; NaHCO_3_, 2.5 g; Resazurin, 1.0 mg; Wolfe’s vitamin solution, 10 ml; and trace metal solution, 10 ml. Wolfe’s vitamin solution contained the following (per litre of distilled water): Biotin, 2.0 mg; Folic acid, 2.0 mg; Pyridoxine-HCl, 10.0 mg; Thiamine-HCl•2H_2_O, 5.0 mg; Riboflavin, 5.0 mg; Nicotinic acid, 5.0 mg; D-Ca-pantothenate, 5.0 mg; Vitamin B_12_, 0.1 mg; *p*-Aminobenzoic acid, 5.0 mg; and Lipoic acid, 5.0 mg. The trace metal solution contained the following (per litre of distilled water): Nitrilotriacetic acid, 1.5 g; MgSO_4_•7H_2_O, 3 g; MnSO_4_•xH_2_O, 0.5 g; NaCl, 1.0 g; FeSO_4_•7H_2_O, 0.1 g; CoSO_4_•7H_2_O, 0.1 g; CaCl_2_•2H_2_O, 0.1 g; ZnSO_4_•7H_2_O, 0.1 g; CuSO_4_•5H_2_O, 0.01 g; AlK(SO_4_)_2_, 0.01 g; H_3_BO_3_, 0.01 g; Na_2_MoO_4_•2H_2_O, 0.01 g; NiCl_2_•6H_2_O, 0.025 g; Na_2_SeO_3_•5H_2_O, 0.3 g; (NH_4_)_2_Ni(SO_4_)_2_•6H_2_O, 2 g; and Na_2_WO_4_•2H_2_O, 10 mg. Headspace gas was exchanged with anoxic N_2_:CO_2_ (80:20, v/v). After sterilisation, the following ingredients were added after filtration: L-cysteine-HCl•H_2_O (0.05%, w/v); Na_2_S•9H_2_O (0.05%, w/v); glucose (0.5%, w/v). When required, 20 mM sodium–potassium phosphate (Na–K-phosphate) buffer was added to the culture medium to adjust the pH to nearly neutral. All cultures were incubated at 30 °C using previously described anaerobic techniques^43^. Bacterial isolation was performed on slant medium prepared in Hungate tubes under anaerobic conditions. The growth of the bacterial isolate was monitored by cell density, as determined by acridine orange staining described below^44^. In all experiments of the decomposition of HAs, the culture medium was supplemented with 0.5% glucose because HSAI-1 could not grow in media supplemented with HAs as the sole carbon source and required glucose as an auxiliary carbon source (Extended data Table 1).

### Acridine orange (AO) direct counting

AO direct counting was used to count bacteria in culture media under a fluorescence microscope^44^. The AO stock solution was prepared by dissolving 100 mg of AO powder (Merck Millipore Co., Germany) in 10 ml of sterile deionized water (1%, w/v); this stock solution was protected from light and stored at 4 °C. The working solution was prepared by diluting the AO stock solution in 10 mM Tris-HCl (pH 7.2) to a final concentration of 0.01%. The AO working solution and 1 × phosphate-buffered saline (PBS) were filter sterilized using a 0.22 μm filter unit (Sartorius, Göttingen, Germany) before use. At each sampling time, a 1 ml sample of each culture medium was obtained using sterilized syringes. Samples containing bacteria were fixed in formalin and then stored at 4 °C overnight. Fixed samples were diluted with 1 × PBS as appropriate and passed through an Isopore membrane filter (pore size 0.22 μm, diameter 47 mm, type GSWP 04700, Millipore, Billerica, MA, USA) to trap bacteria using a vacuum filter unit (SPC filter holders, Shibata Scientific Technology, Ltd., Japan). Bacteria were stained by applying AO solution to the membrane at room temperature in the dark for 3 min. Subsequently, the AO solution was drained by vacuum, and the membrane was rinsed once with 1 × PBS. The membrane filter was sandwiched between a slide and a cover glass. Using epifluorescence microscopy (Olympus BX-51), bacterial cells were enumerated at 1000 × under oil immersion in at least 20–40 randomly chosen microscope fields in triplicate for each sample. The total cell count in each sample was calculated by applying the formula (equation 1)^45^:


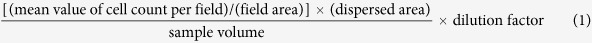


where “field area”, which was 0.012π mm^2^ in our study, was calculated by dividing the field number (FN) by the magnification of the objective (100×); “dispersed area” was the effective size of the region over which the sample was dispersed (*i.e*., the area of the stained region on the Isopore membrane filter, which was 64π mm^2^ in our study); “sample volume” was the volume of bacterial sample applied to the Isopore membrane filter for staining; and “dilution factor” represented the degree to which the formalin-fixed bacterial sample was diluted with 1 × PBS.

### Nucleic acid-based analyses

Genomic DNA of the bacterial isolate was extracted using the Microbial DNA Isolation Kit (MoBio Laboratories, Inc., Carlsbad, CA, USA) according to the manufacturer’s protocol. Subsequently, near full-length 16S rRNA gene fragments (approximately 1,400 bp) were produced by PCR amplification using the universal bacterial primers 27F (5′-AGAGTTTGATCCTGGCTCAG-3′) corresponding to position 8–27 of *E. coli* and 1492R (5′-GGTTACCTTGTTACGACTT-3′) corresponding to position 1510–1492 of *E. coli*^46^. PCR was performed using a PTC-200 DNA Engine Gradient Cycler (MJ Research Science, USA) in a total volume of 20 μL using TaKaRa *Ex Taq* DNA polymerase (TaKaRa Shuzo, Shiga, Japan) in the supplied buffer. The following thermal cycling program was used: initial denaturation at 95 °C for 5 min; 30 cycles each of denaturation at 95 °C for 30 sec, annealing at 55 °C for 30 sec, and extension at 72 °C for 2 min; followed by a final extension at 72 °C for 5 min. Negative controls without DNA template were run for all samples. The PCR products were purified using a QIAquick PCR Purification Kit (Qiagen) and then analysed by SolGent^TM^ Sequencing Service (SolGent Co., Ltd.). The identification of phylogenetic neighbours was carried out by comparing data in the EzTaxon-e program against the database containing strain types with verifiable, published prokaryotic names and representatives of uncultured phylotypes, which was implemented at the EzTaxon-e server^47^. A phylogenetic tree was constructed in MEGA 5.2.2 based on the neighbour-joining method^48^,^49^.

### Synthesis of ^14^C-HA

^14^C-Labelled polycatechol (^14^C-HA), which is an HA-analogue, was synthesised by spontaneous oxidative polymerisation of [U-^14^C]catechol (specific radioactivity, 1.48–1.85 GBq mmol^−1^; radioactivity concentration, 3.7 kBq μl^−1^; American Radiolabeled Chemicals, Inc., Saint Louis, Mo.) in an alkaline solution^5^. [U-^14^C]catechol (1,776 kBq; 1.07 × 10^8^ dpm) and 11 mg of unlabelled catechol were dissolved in 1 ml of NaOH (0.1 N), which also contained Mn^2+^ (final concentration, 2 mM) to enhance the polymerisation process^50^. The reaction mixture was incubated with vigorous stirring at room temperature overnight. ^14^C-HA was precipitated by the addition of 50 μl of 6N HCl, followed by centrifugation. The ^14^C-HA pellet was rinsed with distilled water up to eight times, after which the residual radioactivity level in the supernatant was low and stable (Supplementary Fig. 2). The resulting ^14^C-HA pellet was re-suspended in 1 ml of 0.1N NaOH with a radioactivity of 0.12 × 10^6^ dpm, which contained approximately 0.11% of the original ^14^C-labelled catechol.

### Decomposition experiments with radioactive HA

Decomposition experiments using ^14^C-HA were performed in 3 ml of basal liquid media, as described above, under anaerobic conditions. ^14^C-HA with a radioactivity level of 1,215 dpm was added to each medium, as well as 500 μl of HSAI-1 pre-grown for 7 days. In uninoculated controls, 500 μl of filter-sterilised culture medium containing HSAI-1 was added. Incubation was carried out at 30 °C in the dark. During incubation, 500 μl of headspace gas was obtained using a gas-tight syringe and passed through 2 ml of Carbo-Sorb E (Perkin Elmer Inc.; Waltham, MA, USA), followed by the addition of 2 ml of Permafluor E + (Perkin Elmer Inc.; Waltham, MA, USA) for ^14^CO_2_, and 4 ml of Clearsol I (Nakarai Tesque, Kyoto, Japan) for ^14^C-labelled volatile compounds. After 7 weeks of incubation, ^14^C-HA was dissolved by adding 500 μl of 10N NaOH to the culture media, followed by vigorous shaking. After centrifugation, 500 μl of supernatant was used to determine the soluble radioactivity (^14^C-labelled NaOH-soluble material). The whole pellet after centrifugation was used to determine the insoluble radioactivity (residual ^14^C). A liquid scintillation counter (model LSC-6100; Aloka) was used for radioactivity measurements. Percent mineralisation was calculated after subtracting background counts in the negative controls using the following equation (eq. 2):


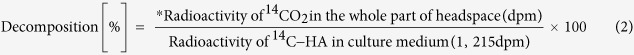


^*^Radioactivity of ^14^CO_2_ in the whole part of headspace (13.6 mL) = Radioactivity of ^14^CO_2_ in 500 μl × 27.2.

### Extraction of HA from culture media

HA was extracted from the culture media according to a previously defined method^7^ with slight modification. Culture media were centrifuged at 20,000 × *g* for 30 min to separate the culture supernatant and cell debris, and then the culture supernatant was transferred to new test tubes. Subsequently, 10N NaOH was added to the culture supernatant to increase the alkalinity of the solution (>pH 12.0) to dissolve the HA completely, and the samples were shaken vigorously on a reciprocal shaker (Type MK201D; Yamato Scientific Co. Ltd., Japan) overnight. The samples were centrifuged at 20,000 × *g* for 20 min to separate the insoluble fraction and supernatant. The resulting supernatant was mixed with 6N HCl to increase the acidity of the solution (<pH 2.0) to precipitate the acid-insoluble HAs. After centrifugation at 20,000 × *g* for 20 min, the HA precipitate was rinsed twice with 0.1N HCl and then once with water. Finally, the rinsed HA precipitate was lyophilized under vacuum overnight. The dried HAs were stored at −20 °C until use.

### Instrumental analyses of HAs

Prior to the instrumental analyses, HAs were extracted from culture media as described above. HPSEC analysis was conducted using a Prominence HPLC (SPD-M20A, Shimadzu, Kyoto, Japan) equipped with a photodiode array detector and a GL-W530 column (Hitachi Hitec, Japan; 10.7 mm I.D. by 300 mm) preceded by a guard column (Hitachi Hitec; 4.0 mm I.D. by 10 mm). According to the manufacturer’s catalogue, the approximate upper exclusion limit was 50,000 Da (calibrated with pullulan). Isocratic elution with a mobile phase mixture of 10 mM Tris-HCl (pH 8.0) and 10 mM NaCl was performed at a flow rate of 1.0 mL min^−1^ at a constant temperature of 40 °C. The column was calibrated using sodium polystyrene sulfonates (1.3–168 kDa). Elution profiles were recorded, and the resulting data were reanalysed with LC Solution software (Shimadzu, Kyoto, Japan). FT-IR analysis was carried out by the Osaka Kankyo Gijutsu Center Co., Ltd. (Osaka, Japan). FT-IR spectra were measured with a Nicolet iS10 FT-IR spectrometer (Thermo Fisher Scientific Inc., USA.) scanning the 4000–400 cm^−1^ range with an average of 128 scans and a spectral resolution of 4 cm^−1^.

### Statistical analysis

Statistical comparisons between the control and test groups were performed using Student’s *t*-test. Differences were considered significant at *P* < 0.05.

## Additional Information

**How to cite this article**: Ueno, A. *et al.* Anaerobic decomposition of humic substances by *Clostridium* from the deep subsurface. *Sci. Rep.* **6**, 18990; doi: 10.1038/srep18990 (2016).

## Supplementary Material

Supplementary Information

Supplementary Dataset

## Figures and Tables

**Table 1:** Mass balance of radioactive carbon.

Sample	% of ^14^C in:		
	^14^CO_2_	Soluble	Insoluble/cell-bound
Control	0.7 ± 1.3	58.5 ± 6.2	40.8 ± 1.3
Strain HSAI-1	6.6 ± 2.2	43.0 ± 2.4	50.4 ± 0.7

Percentage of ^14^C in each fraction after treatment of a synthetic ^14^C-labelled polycatechol analogous to humic acids with *Clostridium* sp. strain HSAI-1. The presented values were obtained at the end of the incubation period (49 days) and are the mean ± standard deviation (*n* = 3).

**Table 2:** Calculated molecular masses of the HA main peaks obtained from the HPSEC analysis.

	Uninoculated control (Da)	HSAI-1 (Da)	*P*-value
Aldrich HA			
0 day	2790.1 ± 15.5	2801.4 ± 12.7	0.242
28 days	2887.3 ± 12.9	2673.1 ± 76.8	<0.001^*^
Koetoi HA			
0 day	3317.9 ± 0.0	3286.2 ± 31.7	0.056
28 days	3229.6 ± 14.0	2824.4 ± 51.8	<0.001^*^

The table shows the mean values of the molecular masses with standard deviations calculated from the retention times obtained from the HPSEC analysis (*n* = 5). Molecular masses were calculated using the equation shown in Supplementary Fig. 4. ^*^Significant differences were detected at 28 days between the uninoculated controls and the HSAI-1 inoculated cultures (*P* < 0.001).
