# Supplementary material for: Anaerobic decomposition of humic substances by Clostridium from the deep subsurface
Source: Sci Rep. 2016 Jan 8;6:18990. doi: 10.1038/srep18990 (PMC4705541; doi:10.1038/srep18990)
Supplement: Supplementary Information [file srep18990-s1.pdf]

Supplementary Information

Anaerobic decomposition of humic substances by *Clostridium* from the deep subsurface

Akio Ueno[1,*], Satoru Shimizu[1], Shuji Tamamura[1], Hidetoshi Okuyama[2], Takeshi Naganuma[3], and Katsuhiko Kaneko[1]

[1] Horonobe Research Institute for the Subsurface Environment, Northern Advancement Centre for Science and Technology, 5-3, Sakae-machi, Horonobe-cho, Teshio-gun, Hokkaido 098-3221, Japan

[2] Graduate School of Environmental Earth Science, Hokkaido University, Sapporo, Hokkaido 060-0810, Japan

[3] Graduate School of Biosphere Science, Hiroshima University, Higashi-Hiroshima 739-8528, Japan

[*] akio.ueno@h-rise.jp

		Cultivation period (days)*			
0.5% Glucose†	Humic acids†	0 ‡	5	8	15
−	−Koetoi HA	$1.2\times10^{7}\pm1.3\times10^{6}$	$9.1\times10^{6}\pm4.1\times10^{5}$	$9.9\times10^{6}\pm7.6\times10^{5}$	$6.6\times10^{6}\pm 4.8\times10^{5}$
	＋Koetoi HA	$1.3\times10^{7}\pm4.9\times10^{5}$	$1.1\times10^{7}\pm8.3\times10^{5}$	$9.3\times10^{6}\pm4.6\times10^{5}$	$8.3\times10^{6}\pm1.8\times10^{6}$
＋	−Koetoi HA	$1.4\times10^{7}\pm1.1\times10^{6}$	$5.1\times10^{8}\pm3.9\times10^{7}$	$6.1\times10^{8}\pm2.4\times10^{7}$	$5.2\times10^{8}\pm1.6\times10^{7}$
	＋Koetoi HA	$1.4\times10^{7}\pm2.4\times10^{6}$	$4.9\times10^{8}\pm1.6\times10^{7}$	$5.1\times10^{8}\pm 8.5\times10^{6}$	$6.7\times10^{8}\pm1.1\times10^{8}$
−	−Aldrich HA	$1.3\times10^{7}\pm1.5\times10^{6}$	$7.6\times10^{6}\pm4.9\times10^{5}$	$6.4\times10^{8}\pm2.6\times10^{5}$	$6.9\times10^{6}\pm5.5\times10^{5}$
	＋Aldrich HA	$1.5\times10^{7}\pm3.0\times10^{6}$	$1.1\times10^{7}\pm9.3\times10^{4}$	$8.9\times10^{6}\pm8.4\times10^{5}$	$9.5\times10^{6}\pm7.9\times10^{5}$
＋	−Aldrich HA	$1.3\times10^{7}\pm1.3\times10^{6}$	$5.3\times10^{8}\pm1.4\times10^{7}$	$6.1\times10^{8}\pm3.9\times10^{7}$	$4.5\times10^{8}\pm8.4\times10^{7}$
	＋Aldrich HA	$1.5\times10^{7}\pm1.0\times10^{6}$	$5.3\times10^{8}\pm6.9\times10^{7}$	$6.1\times10^{8}\pm6.5\times10^{7}$	$5.7\times10^{8}\pm4.4\times10^{7}$

Supplementary Table 1. Cell density of *Clostridium* sp. strain HSAI-1. *Unit of cell density: cells ml^{-1}. The total cell count in each sample was calculated by acridine orange (AO) direct counting described in Methods section. Cell densities are presented as the mean ± standard deviation (n = 3). †+, added; −, not added. ‡No significant differences were detected between any experimental groups at day 0.

	Absorption bands (cm^{-1})	Near 3410~3420	Near 3290~3300	Near 3070	Near 2930	Near 1710	Near 1650 (1620)	Near 1540	Near 1450	Near 1230~1240	Near 1035
Humic acids*	Proposed assignment†	H-bonded OH groups	N-H stretching vibration	Aromatics	Aliphatic C-H stretching vibrations in CH_2 and/or CH_3 groups	C=O stretching in COOH, aldehyde and ketones	Amide I (C=O in peptide linkage)	Amide II (N-H in peptide linkage)	C-H deformation of aliphatic CH_2 and CH_3 groups	C-O stretching vibration and O-H deformation of COOH groups	C-O stretching and O-H deformation in alcoholic and polysaccharide
Aldrich HAs	(a) Control	+	–	–	+	+	+	–	–	+	–
	(b) HSAI-1	–	+	+	+	–	+	+	+	+	–
Koetoi HAs	(c) Control	+	–	–	+	+	+	–	–	+	+
	(d) HSAI-1	–	+	+	+	–	+	+	+	+	–

Supplementary Table 2. Presence and absence of infrared absorption bands of Aldrich and Koetoi HAs in FT-IR spectra.

*+: A clear absorption band was observed.; –: The absorption band was very weak or not detected.

† The proposed assignment has been reported previously[5,6,34,51,52].

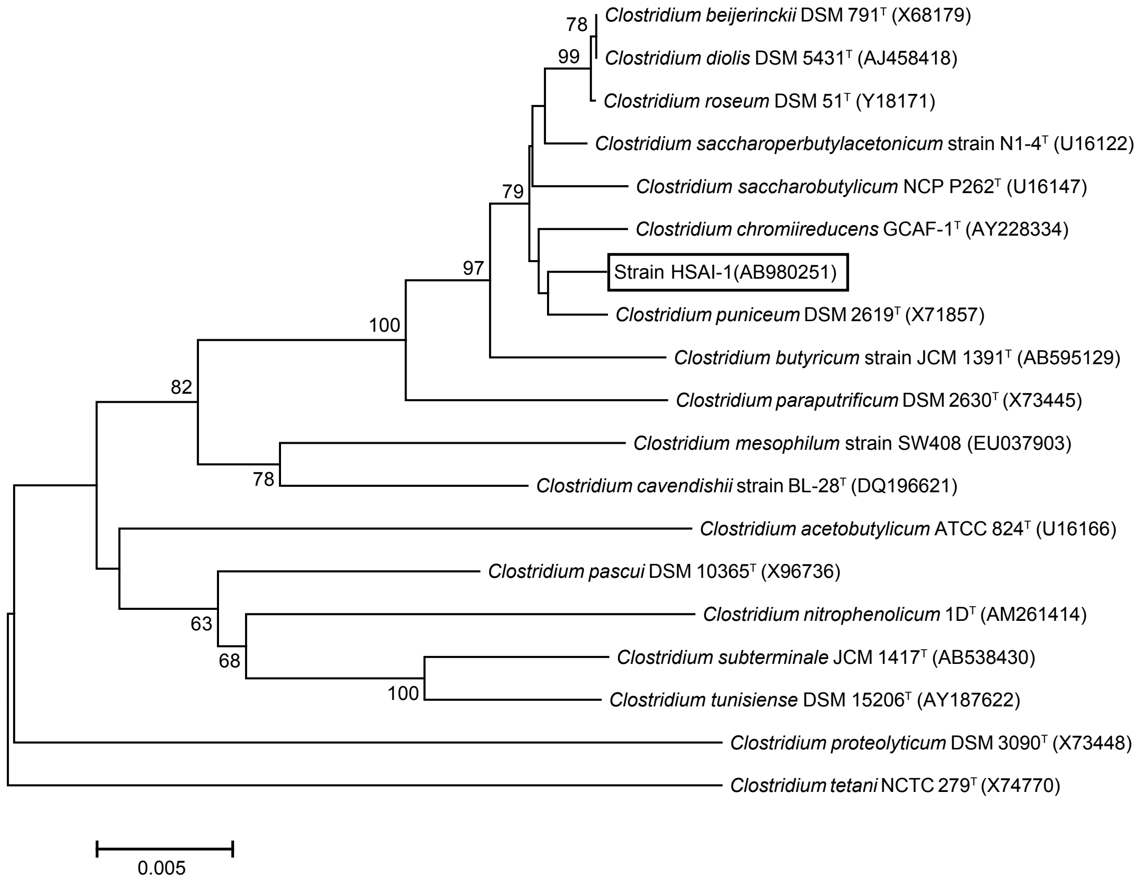

Supplementary Figure 1. Phylogenetic analysis of the bacterial isolate based on the nearly full-length 16S rRNA gene sequence (approximately 1,400 bp). The 16S rRNA gene sequence of *Clostridium tetani* strain NCTC 279^{T} (X74770) was used as the outgroup. The numbers at the nodes are bootstrap values derived from 1,000 replicates. The accession numbers are shown in parentheses. The bar represents 0.005 nucleotide changes per site. Bacterial strain HSAI-1 (Horonobe anaerobic subsurface isolates-1) is indicated with a box.

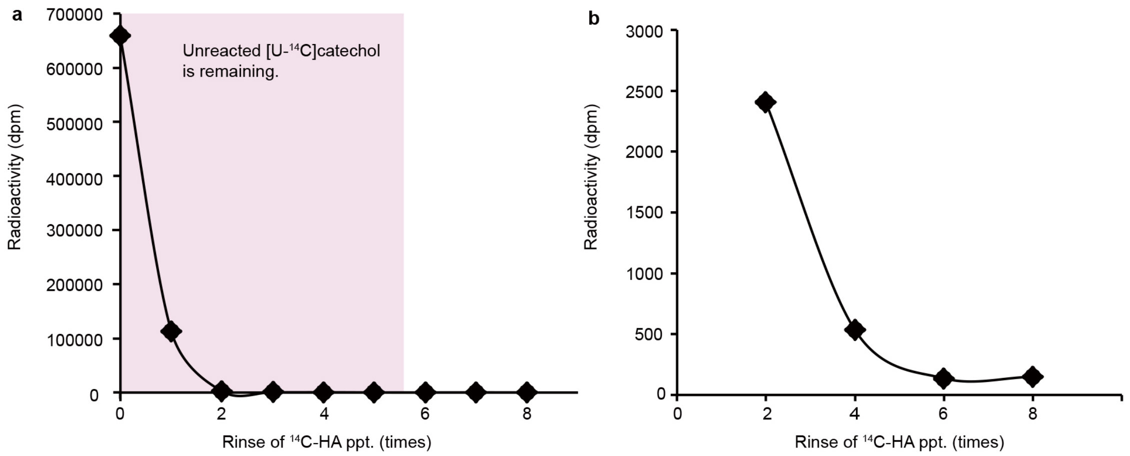

Supplementary Figure 2. Residual radioactivity of unreacted [^{14}C]catechol in the supernatant. **(a)** Unreacted [U-^{14}C]catechol in the supernatant was monitored while rinsing the pellet of ^{14}C-labelled catechol (^{14}C-HA), which is an HA analogue, via liquid scintillation counting after synthesizing ^{14}C-HA. (**b)** Enlarged version of the left figure. The amount of residual [U-^{14}C]catechol in the supernatant decreased by the 6th rinsing and then remained constant. We used the ^{14}C-HA pellet after the 8th rinsing in the tracer experiment. dpm, decays per minute, corrected from the counts per minute (cpm).

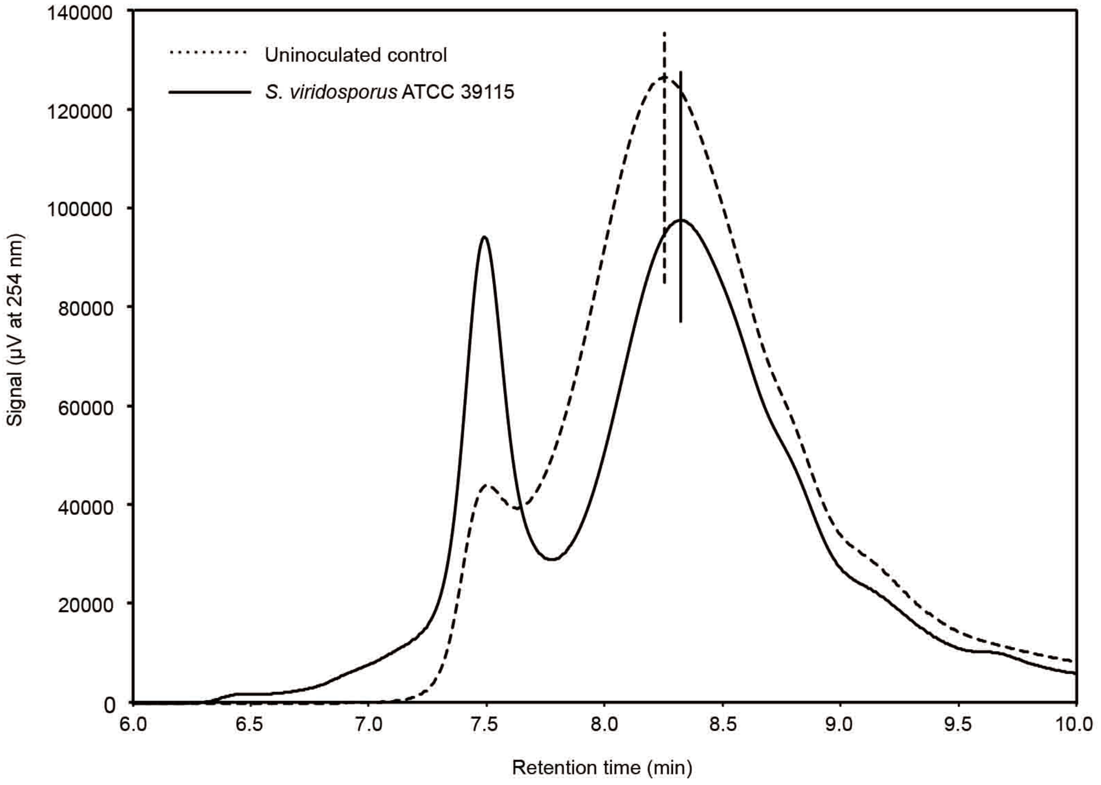

Supplementary Figure 3. Chromatograms of HAs extracted from the culture media of *Streptomyces viridosporus* ATCC 39115. HAs extracted from the culture media of the HA-degrading actinomycete *S. viridosporus* ATCC 39115 were analysed via high- performance size exclusion chromatography (HPSEC). Representative data (*n* = 5) are shown in this figure. Shown are uninoculated control (dotted line) and *S. viridosporus* ATCC 39115-inoculated (solid line) cultures. The tops of the main peaks with retention times ranging from 8.2 to 8.5 min are indicated with vertical lines: a dotted line for the uninoculated controls and a solid line for the inoculated cultures.

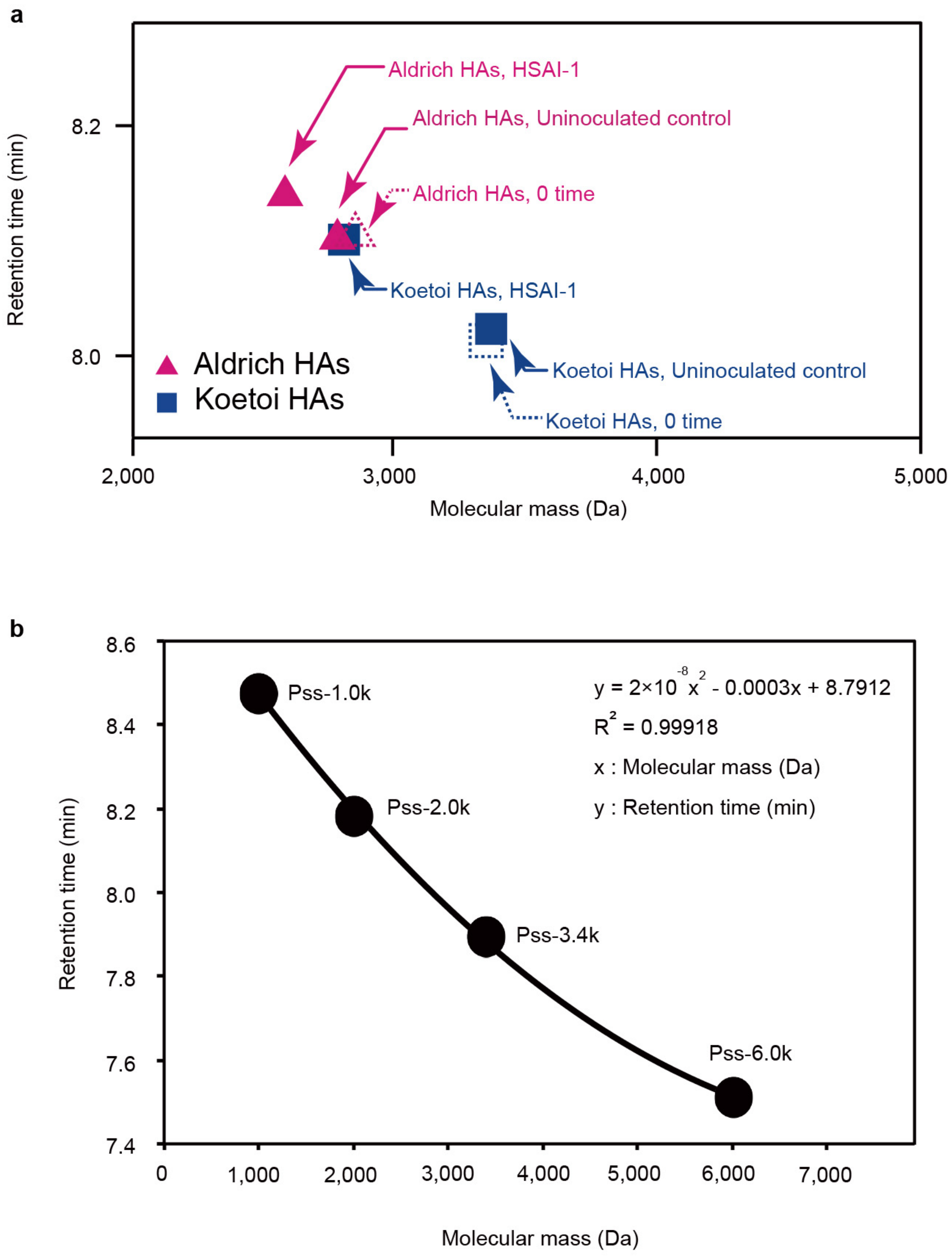

Supplementary Figure 4. The molecular mass of HAs was reduced following HSAI-1 cultivation. (a) The retention time of the main HA peak is plotted, and the molecular mass is calculated by the equation obtained from **(b)** the regression line of

polystyrene sulfonate (Pss) standards (black circles). Notably, the molecular mass of the main HA peak in the HSAI-1-inoculated culture was lower than that mass of the uninoculated control. The values at the beginning of incubation (day 0) are indicated with dotted marks.

Supplementary References

51. Alex, S., Savoie, R., Corbeil, M.-C. & Beauchamp, A. L. Complexation of glycylglycine by the methylmercury cation: a vibrational spectroscopy and X-ray diffraction study. *Can. J. Chem.* **64**, 148-157 (1986).
52. Filip, Z., Pecher, W. & Berthelin, J. Microbial utilization and transformation of humic acid-like substances extracted from a mixture of municipal refuse and sewage sludge disposed of in a landfill. *Environ. Pollut.* **109**, 83-89 (2000).
